# Depression and anxiety among patients undergoing dialysis and kidney transplantation: a cross-sectional study

**DOI:** 10.1590/1516-3180.2018.0272280119

**Published:** 2019-07-15

**Authors:** Daniela Cristina Sampaio de Brito, Elaine Leandro Machado, Ilka Afonso Reis, Lilian Pires de Freitas do Carmo, Mariangela Leal Cherchiglia

**Affiliations:** I MSc. Psychologist and Doctoral Student, Research Group on Economy and Health, Department of Preventive and Social Medicine, Universidade Federal de Minas Gerais (UFMG), Belo Horizonte (MG), Brazil.; II MD, PhD. Psychologist and Professor, Department of Preventive and Social Medicine, Universidade Federal de Minas Gerais (UFMG), Belo Horizonte (MG); and Researcher, Research Group on Economy and Health, Department of Preventive and Social Medicine, Universidade Federal de Minas Gerais (UFMG), Belo Horizonte (MG), Brazil.; III MD, PhD. Statistician and Professor, Department of Statistics, Universidade Federal de Minas Gerais (UFMG), Belo Horizonte (MG); and Researcher, Research Group on Economy and Health, Department of Preventive and Social Medicine, Universidade Federal de Minas Gerais (UFMG), Belo Horizonte (MG), Brazil.; IV MD, PhD. Doctor and Professor, Department of Internal Medicine, Universidade Federal de Minas Gerais (UFMG), Belo Horizonte (MG), Brazil.; V MD, PhD. Doctor and Professor, Department of Preventive and Social Medicine, Universidade Federal de Minas Gerais (UFMG), Belo Horizonte (MG); and Coordinator, Research Group on Economy and Health, Department of Preventive and Social Medicine, Universidade Federal de Minas Gerais (UFMG), Belo Horizonte (MG), Brazil

**Keywords:** Depression, Anxiety, Quality of life, Kidney failure, chronic, Renal replacement therapy

## Abstract

**BACKGROUND:**

Depression and anxiety are the most prevalent psychological disorders among end-stage renal disease patients and are associated with various conditions that result in poorer health outcomes, e.g. reduced quality of life and survival. We aimed to investigate the prevalences of depression and anxiety among patients undergoing renal replacement therapy.

**DESIGN AND SETTING:**

Cross-sectional study in Belo Horizonte, Brazil.

**METHODS:**

Patients’ depression and anxiety levels were assessed using the Beck Inventory. The independent variables were the 36-Item Short-Form Health Survey (SF-36), Charlson Comorbidity Index and Global Subjective Assessment, along with sociodemographic and clinical characteristics.

**RESULTS:**

205 patients were included. Depression and anxiety symptoms were detected in 41.7% and 32.3% of dialysis patients and 13.3% and 20.3% of transplantation patients, respectively. Lower SF-36 mental summary scores were associated with depression among transplantation patients (odds ratio, OR: 0.923; 95% confidence interval, CI: 0.85-0.99; P = 0.03) and dialysis patients (OR: 0.882; 95% CI: 0.83-0.93; P ≤ 0.001). Physical component summary was associated with depression among dialysis patients (OR: 0.906; 95% CI: 0.85-0.96; P = 0.001). Loss of vascular access (OR: 3.672; 95% CI: 1.05-12.78; P = 0.04), comorbidities (OR: 1.578; 95% CI: 1.09-2.27; P = 0.01) and poorer SF-36 mental (OR: 0.928; 95% CI: 0.88-0.97; P = 0.002) and physical (OR: 0.943; 95% CI: 0.89-0.99; P = 0.03) summary scores were associated with anxiety among ­dialysis patients.

**CONCLUSIONS:**

Depression and anxiety symptoms occurred more frequently among patients undergoing dialysis. Quality of life, comorbidities and loss of vascular access were associated factors.

## INTRODUCTION

Despite advancements in renal replacement therapies and increased survival, patients still face several physical, psychological and social limitations as consequences of chronic kidney disease and treatment complexity.^1^^,^^2^ The daily struggle with end-stage renal disease symptoms and related comorbidities, along with the need to cope with psychosocial stressors, directly impacts patients’ quality of life and mental health.^3^^,^^4^

Depression and anxiety are considered to be the most common end-stage renal disease-related psychological disorders, with higher prevalence and incidence rates in this population than those in the general population.^5^^,^^6^^,,^^7^^,^^8^^,^^9^^,^^10^ According to the World Health Organization, the estimated global prevalence rates of depression and anxiety in 2015 were 4.4% and 3.6%, respectively, with an increase in reported cases of 18% between 2005 and 2015.^11^ The anxiety and depression rates that have been estimated among end-stage renal disease patients are not accurate: they range from 0 to 100%, depending on the diagnostic criteria, assessment tool and population characteristics.^12^ A systematic review of 55 studies revealed prevalence rates of 38% and 27% for anxiety and depression, respectively, among end-stage renal disease patients.^13^

The high frequency and impact of affective symptoms in nephrology practice have led the research community to devote increasing attention to depression and anxiety over the last few years.^7^ In end-stage renal disease, these mental disorders are associated with various conditions that lead to poorer health outcomes, with direct impacts on patients’ quality of life and survival.^14^^,^^15^^,^^16^^,^^17^^,^^18^^,^^19^^,^^20^^,^^21^^,^^22^^,^^23^ Anxiety and depression are also associated with unhealthy forms of behavior, such as alcohol and tobacco use, poor eating habits, sedentary lifestyle and non-compliance with treatment.^24^ Thesefactors translate into increased risks of clinical events and the need for emergency services, thus resulting in higher healthcare costs.^25^^,^^26^

Given the need for better understanding of affective disorders and associated factors in end-stage renal disease, the present study set out to 1) investigate the prevalence of depression and anxiety among patients undergoing different types of renal replacement therapy and 2) investigate the factors associated with the presence and severity of depression and anxiety symptoms. Kidney transplantation is believed to favor a better clinical condition and a daily routine that is more active and less dependent on the restrictions imposed by dialysis. Our hypothesis was that dialysis patients would present higher prevalence of depression and anxiety symptoms than a group of transplantation patients.

## METHODS

### Study design

**Figure 1.**
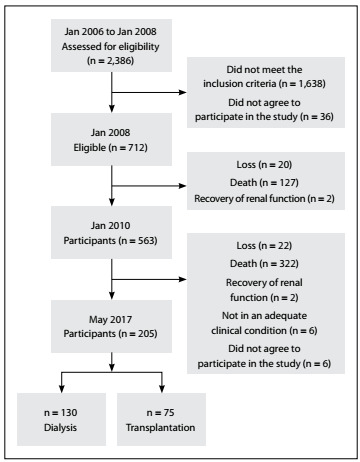
Flowchart of the study design.

This investigation consisted of a cross-sectional follow-up study on participants in a cohort that had been established in 2006. Thecohort included patients who were undergoing renal replacement therapy at 10 public dialysis services funded by the Brazilian public healthcare system in Belo Horizonte, Minas Gerais, Brazil^27^ (Figure 1).

### Participants

The initial cohort included all patients aged 18 years or over who started to undergo dialysis between January 1, 2006, and January 1, 2008, with a minimum of three months of treatment and no previous history of kidney transplantation. A total of 748 out of 2,386 patients met the selection criteria, and 36 of these patients refused to participate. Therefore, 712 patients formed the initial sample and were followed up over a non-concurrent or retrospective period (January 2006 to January 2008) and a concurrent or prospective period (January 2006 to May 2017).

The participants in the present cross-sectional study were patients who were undergoing dialysis or were surviving transplantation between February and May 2017, and whose physical condition and cognitive ability were sufficient for them to be able to complete the questionnaires. Patients who refused to participate, those who recovered their renal function and those who were referred for treatment elsewhere were excluded.

### Ethical considerations

The Institutional Review Board (IRB) at the Federal University of Minas Gerais (UFMG) approved this study (under procedural no. 1.747.336), and all participants signed an informed consentform.

### Measurements

Sociodemographic and clinical data were collected during structured interviews or were extracted from the medical records maintained by the participating units. Comorbidities were measured using the Charlson Comorbidity Index (CCI): this is a scoring system comprising 19 comorbidity items with assigned weights ranging from one to six, such that higher summed scores correspond to clinical conditions of greater severity.^28^ Nutritionalstatus was determined using the Subjective Global Assessment (SGA), which is a method that categorizes patients as well-nourished, suspected of being malnourished or severely malnourished, based on features of their physical examinations and clinical histories.^29^

Additional groups of covariates were selected as follows:

- sociodemographic: sex, age, ethnic group, marital status, religion, level of education, occupation and income.
- clinical: length of time undergoing renal replacement therapies; whether listed in transplantation waiting lists; occurrence of graft loss after renal transplantation; current vascular access; number of visits and admissions; and different types of medications used.
- life habits: social activities (participation in unions, associations, organizations and diverse groups, such as seniors’, ­men’s/­women’s, religious or political groups); recreational activities (attendance of parties, clubs, soccer stadiums and gatherings with family/friends); and tobacco and alcohol use. We defined alcohol use as consumption of five or more drinks (for males) or four or more drinks (for females) on a single occasion within the last 30 days.
- healthcare service(type of renal replacement therapy facility and travel time): for classification purposes, the facilities were grouped according to indicators of capacity to handle cases of increasing levels of complexity (graded from one to three), through a calculation using principal component analysis (PCA). The characteristics associated with the level of complexity with minimal variability between facilities included the type of service (outpatient or inpatient), teaching activities (yes or no) and kidney transplantation service availability (yes or no).

Depression and anxiety symptoms were assessed using the Beck Depression Inventory (BDI) and Beck Anxiety Inventory (BAI), validated for the Brazilian population.^30^ The BDI and BAI are questionnaires consisting of 21 depression-related and 21 anxiety-related items to evaluate the presence and severity of symptoms over the course of the last week. Items are scored from 0 to 3, with a total summed score ranging from 0 to 63 points. The scores indicating depression and anxiety are those greater than the cutoff of 11 and 10 points, respectively. Higher scores correspond to more severe symptoms, and the levels of depression and anxiety are graded as minimal or absent, mild, moderate or severe. TheBAI and BDI tests were applied in accordance with Resolution 9 of the Brazilian Federal Psychology Council.^31^

The quality-of-life assessment was based on the Portuguese version of the 36-item Short-Form Health Survey (SF-36).^32^ This instrument comprises eight domains (physical functioning, role-physical, bodily pain, general health status, vitality, social functioning, role-emotional and mental health) and two summary measurements (physical and mental component summaries). Its scores can range from 0 to 100, and scores closer to 100 indicate better quality of life.

The patients were interviewed by trained health-related undergraduate students who were participating in the research project. The interviews were conducted over the course of dialysis sessions (hemodialysis patients) or during follow-up visits (peritoneal dialysis and kidney transplantation patients).

### Statistical analysis

Descriptive statistics were produced, based on frequencies for categorical variables, or on means ± standard deviations (SDs) for quantitative variables with normal distribution or medians for those with non-normal distribution. The non-paired Student t test and the Mann-Whitney test were used to compare normally and non-normally distributed quantitative variables, respectively. Pearson’s chi-square test and Fisher’s exact test were used to compare categorical variables. Risk factors for depression and anxiety and symptom severity were analyzed using age- and sex-adjusted multivariate logistic regression models. For the logistic regression analysis, all variables that showed a significance level of 0.20 or lower were tested and only those with a significance level of 0.05 or lower were presented in the final model. Statistical analyses were performed using SPSS version 16.0.

## RESULTS

### Patient characteristics

This cross-sectional analysis included 205 patients: 130 of them were on dialysis and 75 of them had undergone transplantation. A majority of the patients were male (52.7%), and many were married or in a *de facto* relationship (56.1%). The mean age was 54.5 years (SD = 12.7). In addition, many of the patients had not completed elementary education (58.2%), most did not have a job (78.5%) and most were living on some form of government-provided benefit (79.8%) (Tables 1and2).

**Table 1.** Sociodemographic and clinical characteristics of transplantation and dialysis patients according to symptoms of depression

	Total (n = 205)	Transplantation (n = 75)		P-value	Dialysis (n = 130)		P-value
		BDI ≤ 11 (n = 65)	BDI ≥ 12 (n = 10)		BDI ≤ 11 (n = 53)	BDI ≥ 12 (n = 77)	
	M ± SD/n (%)	M ± SD/n (%)	M ± SD/n (%)		M ± SD/n (%)	M ± SD/n (%)	
Sociodemographic variables							
Age	54.5 ± 12.7	51.9 ± 11.3	50.7 ± 9.1	0.75	56.5 ± 13.2	55.6 ± 13.1	0.70
Sex (female)	97 (47.3)	24 (36.9)	9 (90.0)	0.001	41 (55.4)	21 (39.6)	0.07
Skin color (brown/black)	148 (75.5)	44 (72.1)	6 (60.0)	0.46	51 (70.8)	45 (90.0)	0.01
Religion (yes)	191 (93.6)	58 (90.6)	9 (90.0)	1	72 (97.3)	49 (92.5)	0.23
Married/*de facto* relationship	115 (56.1)	46 (70.8)	3 (30.0)	0.02	41 (55.4)	25 (47.2)	0.36
Schooling							
Up to 9 years	114 (58.2)	32 (50.0)	5 (50.0)	0.64	42 (60.9)	34 (66.7)	0.43
10 to 12 years	58 (29.6)	21 (32.8)	4 (40.0)		18 (26.1)	14 (27.5)	
Over 12 years	24 (12.2)	11 (17.2)	1 (10.0)		9 (13.0)	3 (5.9)	
Job (yes)	44 (21.5)	24 (36.9)	2 (20.0)	0.47	11 (14.9)	7 (13.2)	0.79
Source of income							
Work	37 (20.2)	21 (37.5)	2 (25.0)	0.70	10 (14.9)	4 (8.2)	0.26
Benefits	146 (79.8)	35 (62.5)	6 (75.0)		57 (85.1)	45 (91.8)	
Clinical variables							
Time on dialysis (months)	120.1 ± 8.1	44.5 ± 39.6	47.88 ± 33.9	0.82	120.65 ± 8.30	119.38 ± 8.10	0.40
Time since Tx (months)	77.6 ± 38.0	77.0 ± 38.4	81.6 ± 36.9	0.72	- -	- -	- -
Waiting list for Tx (yes)	89 (43.4)	- -	- -	- -	51 (68.9)	37 (69.8)	0.9
Graft loss after renal Tx	17 (8.2)	- -	- -	- -	9 (12.2)	8 (15.1)	0.63
Vascular access (fistula)	106 (51.7)	- -	- -	- -	57 (77.0)	46 (86.8)	0.34
Loss of vascular access	26 (20.2)	- -	- -	- -	13 (17.8)	13 (24.5)	0.35
CCI	2.00	2.00	1.00	0.67	1.00	2.00	0.01
Visits	122 (59.5)	33 (50.8)	7 (70.0)	0.32	40 (54.1)	40 (75.5)	0.01
Hospital admissions	84 (41.0)	26 (40.0)	5 (50.0)	0.73	25 (33.8)	27 (50.9)	0.05
Number of medications	6.0	6.0	6.0	0.44	5.0	6.0	0.89
SGA (well-nourished)	187 (92.1)	63 (96.9)	10 (100)	1	68 (91.9)	45 (84.9)	0.21
Service unit							
RRT unit							
Group 1	35 (17.2)	0	0	1	19 (26.0)	16 (30.2)	0.74
Group 2	81 (39.9)	25 (38.5)	3 (33.3)		29 (39.7)	22 (41.5)	
Group 3	87 (42.9)	40 (61.5)	6 (66.7)		25 (34.2)	15 (28.3)	
Distance to unit (min)	50.0	63.1 ± 39.4	54.4 ± 50.00	0.28	50.0	40.0	0.02
Habits							
Smoking	16 (7.8)	3 (4.7)	1 (10.0)	0.44	8 (10.8)	4 (7.5)	0.53
Alcoholic drinks	26 (12.7)	10 (15.4)	0	0.34	10 (13.5)	6 (11.3)	0.71
Recreational activities (yes)	141 (68.8)	52 (80.0)	8 (80.0)	1	44 (59.5)	36 (67.9)	0.33
Social activities (yes)	85 (41.5)	36 (55.4)	5 (50.0)	1	26 (35.1)	18 (34.0)	0.89
SF-36 scores							
Physical functioning	59.6 ± 28.2	74.0 ± 24.8	68.0 ± 21.6	0.46	57.2 ± 25.4	43.1 ± 28.0	0.005
Role-physical	50.1 ± 43.4	71.9 ± 39.6	42.5 ± 45.7	0.08	49.3 ± 41.7	25.4 ± 34.4	0.001
Bodily pain	57.4 ± 28.1	69.1 ± 23.5	47.5 ± 17.9	0.001	62.1 ± 28.3	38.0 ± 24.2	< 0.001
General health status	57.0 ± 24.4	70.4 ± 20.9	58.2 ± 26.2	0.1	61.1 ± 17.8	34.2 ± 20.9	< 0.001
Vitality	57.2 ± 22.1	66.6 ± 19.2	47.0 ± 27.8	0.05	61.8 ± 16.7	40.6 ± 21.7	< 0.001
Social functioning	76.1 ± 27.0	87.6 ± 17.8	70.0 ± 40.4	0.02	81.4 ± 19.5	55.5 ± 31.4	< 0.001
Role-emotional	66.4 ± 43.1	88.7 ± 26.5	53.4 ± 47.6	0.001	69.8 ± 42.8	36.5 ± 42.4	< 0.001
Mental health	70.7 ± 19.7	76.2 ± 19.4	51.2 ± 10.7	0.000	77.7 ± 13.4	57.5 ± 20.4	< 0.001
Physical component summary	39.1 ± 11.0	45.4 ± 9.4	41.7 ± 10.2	0.25	38.7 ± 9.7	31.3 ± 9.9	< 0.001
Mental component summary	50.1 ± 11.1	54.1 ± 8.6	41.0 ± 13.8	0.000	53.6 ± 7.8	41.8 ± 12.0	< 0.001

BDI = Beck Depression Inventory; CCI = Charlson Comorbidity Index; SGA = Subjective Global Assessment; SF-36 = 36-item Short-Form Health Survey; M = mean; SD = standard deviation; RRT = renal replacement therapy; Tx = transplantation. BDI>11= symptoms of depression. Loss of vascular access: number of losses of vascular access in the last 12 months. Visits and hospital admissions:number of patients with at least one visit/admission in the last 12 months. Continuous variables with normal distribution (Shapiro-Wilk normality test) were summarized using the mean ± standard deviation (SD) and were compared using the t test. For other quantitative variables, the median was used as a summary measurement, and the Mann-Whitney test was used for comparisons within the group.

**Table 2.** Sociodemographic and clinical characteristics of transplantation and dialysis patients according to symptoms of anxiety

	Total (n = 205)	Transplantation (n = 75)		P-value	Dialysis (n = 130)		P-value
		BAI ≤ 10 (n = 60)	BAI ≥ 11 (n = 15)		BAI ≤ 10 (n = 89)	BAI ≥ 11 (n = 41)	
	M ± SD/n (%)	M ± SD/n (%)	M ± SD/n (%)		M ± SD/n (%)	M ± SD/n (%)	
Sociodemographic variables							
Age	54.5 ± 12.7	51.9 ± 11.1	51.3 ± 11.6	0.86	56.7 ± 13.7	54.4 ± 11.3	0.36
Sex (female)	97 (47.3)	22 (37.3)	11 (73.3)	0.01	38 (44.2)	23 (56.1)	0.20
Skin color (brown/black)	148 (75.5)	39 (70.9)	11 (73.3)	1	61 (73.5)	36 (92.3)	0.01
Religion (yes)	191 (93.6)	52 (89.7)	14 (93.3)	1	82 (95.3)	39 (95.1)	1
Married/*de facto* relationship	115 (56.1)	40 (67.8)	8 (53.3)	0.29	43 (50.0)	23 (56.1)	0.52
Schooling							
Up to 9 years	114 (58.2)	27 (46.6)	9 (60.0)	0.64	50 (61.7)	25 (65.8)	0.84
10 to 12 years	58 (29.6)	21 (36.2)	4 (26.7)		22 (27.2)	10 (26.3)	
Over 12 years	24 (12.2)	10 (17.2)	2 (13.3)		9 (11.1)	3 (7.9)	
Job (yes)	44 (21.5)	21 (35.6)	5 (33.3)	0.87	13 (15.1)	5 (12.2)	0.65
Source of income							
Work	37 (20.2)	19 (37.3)	4 (33.3)	1	9 (11.5)	5 (13.2)	0.77
Benefits	146 (79.8)	32 (62.7)	8 (66.7)		69 (88.5)	33 (86.8)	
Clinical variables							
Time on dialysis (months)	120.1 ± 8.1	42.3 ± 39.8	51.3 ± 33.1	0.45	119.6 ± 8.1	121.1 ± 8.3	0.34
Time since Tx (months)	77.6 ± 38.0	80.0 ± 38.8	72.0 ± 33.4	0.46	- -	- -	- -
Waiting list for Tx (yes)	89 (43.4)	- -	- -	- -	62 (72.1)	26 (63.4)	0.32
Graft loss after renal Tx	17 (8.2)	- -	- -	- -	14 (16.3)	3 (7.3)	0.21
Vascular access (fistula)	106 (51.7)	- -	- -	- -	69 (80.2)	35 (85.4)	0.76
Loss of vascular access	26 (20.2)	- -	- -	- -	12 (14.1)	14 (34.1)	0.009
CCI	2.00	2.00	2.00	0.01	1.00	2.00	0.001
Visits	122 (59.5)	29 (49.2)	11 (73.3)	0.14	51 (59.3)	29 (70.7)	0.21
Hospital admissions	84 (41.0)	25 (42.4)	5 (33.3)	0.52	29 (33.7)	24 (58.5)	0.008
Number of medications	6.0	7.0	6.0	0.32	6.0	5.5	0.50
SGA (well-nourished)	187 (92.1)	57 (96.6)	15 (100)	1	80 (93.0)	33 (80.5)	0.06
Service unit							
RRT unit							
Group 1	35 (17.2)	0	0	0.13	24 (28.2)	11 (26.8)	0.27
Group 2	81 (39.9)	25 (43.1)	3 (20.0)		30 (35.3)	20 (48.8)	
Group 3	87 (42.9)	33 (56.9)	12 (80.0)		31 (36.5)	10 (24.4)	
Distance to unit (min)	50.0	60.2 ± 37.1	60.7 ± 56.2	0.9	47.5	40.0	0.09
Habits							
Smoking	16 (7.8)	3 (5.2)	1 (6.7)	1	8 (9.3)	4 (9.8)	1
Alcoholic drinks	26 (12.7)	10 (16.9)	0	0.21	12 (14.0)	4 (9.8)	0.50
Recreational activities (yes)	141 (68.8)	46 (78.0)	13 (86.7)	0.72	54 (62.8)	27 (65.9)	0.73
Social activities (yes)	85 (41.5)	32 (54.2)	8 (53.3)	0.95	27 (31.4)	17 (41.5)	0.26
SF-36 scores							
Physical functioning	59.6 ± 28.2	75.1 ± 23.3	67.0 ± 19.8	0.25	54.2 ± 27.0	45.6 ± 27.4	0.09
Role-physical	50.1 ± 43.4	75.0 ± 38.2	41.6 ± 44.9	0.01	45.0 ± 41.5	28.0 ± 36.3	0.02
Bodily pain	57.4 ± 28.1	70.5 ± 24.3	52.0 ± 11.8	0.006	61.2 ± 27.2	33.4 ± 24.0	< 0.001
General health status	57.0 ± 24.4	70.4 ± 21.5	62.4 ± 23.8	0.21	55.3 ± 21.6	38.8 ± 22.7	< 0.001
Vitality	57.2 ± 22.1	66.6 ± 20.0	54.6 ± 25.0	0.10	57.1 ± 20.3	44.8 ± 22.0	0.004
Social functioning	76.1 ± 27.0	87.5 ± 18.2	76.6 ± 34.9	0.1	79.1 ± 23.4	53.3 ± 29.3	< 0.001
Role-emotional	66.4 ± 43.1	89.2 ± 26.5	66.7 ± 43.6	0.01	64.7 ± 43.4	38.1 ± 45.0	0.002
Mental health	70.7 ± 19.7	77.4 ± 19.4	56.2 ± 15.1	0.000	74.4 ± 17.2	58.8 ± 19.5	< 0.001
Physical component summary	39.1 ± 11.0	46.0 ± 9.9	41.3 ± 7.5	0.09	37.5 ± 10.0	31.8 ± 10.3	0.004
Mental component summary	50.1 ± 11.1	54.2 ± 8.9	45.7 ± 13.2	0.004	51.8 ± 9.9	42.4 ± 11.7	< 0.001

BAI = Beck Anxiety Inventory; CCI = Charlson Comorbidity Index; SGA = Subjective Global Assessment; SF-36 = 36-Item Short-Form Health Survey; M = mean; SD = standard deviation; RRT = renal replacement therapy; Tx = transplantation. BAI > 11 = symptoms of anxiety. Loss of vascular access: number of losses of vascular access in the last 12 months. Visits and hospital admissions: number of patients with at least one visit/admission in the last 12 months. Continuous variables with normal distribution (Shapiro-Wilk normality test) were summarized using the mean ± standard deviation (SD) and were compared using the t test. For other quantitative variables, the median was used as a summary measurement, and the Mann-Whitney test was used for comparisons within the group.

The dialysis and transplantation patients differed in relation to the following: capacity to perform recreational activities within daily living (62.3% and 80.0%, respectively); capacity to work (13.8% and 34.7%, respectively); benefits as the major source of income (88.2% and 64.1%, respectively); and good nutritional status (89.1% and 97.3%, respectively). Overall, symptoms of both depression and anxiety were observed in 31.2% and 27.9%, respectively, of the sample studied. Moreover, depression affected 41.7% and 13.3% of the dialysis and transplantation patients, respectively, whereas anxiety affected 32.3% and 20.3% of the dialysis and transplantation patients, respectively (Tables 1and2).

### Characteristics of the patients with depressive symptoms

Univariate analysis revealed that most of the transplantation patients with depressive symptoms were women (P = 0.001), most were not married or in a *de facto* relationship (P = 0.02) and many had lower scores in the SF-36 domains of bodily pain (P=0.001), social functioning (P = 0.02), role-emotional (P=0.001) and mental health (P ≤ 0.001), and in the mental component summary (P ≤ 0.001) (Table 1). Compared with non-depressive dialysis patients, depressed dialysis patients mostly had brown/black skin color (P = 0.01), presented more comorbidities, as shown by a higher CCI (P = 0.01), had had higher numbers of visits (P = 0.01) over the last 12 months, had shorter travel times to the healthcare service (P = 0.02) and had lower SF-36 scores (Table 1).

Logistic regression revealed associations between the mental component summary of the SF-36 and depression among transplantation patients (OR = 0.923; P = 0.03) and dialysis patients (OR=0.882; P ≤ 0.001). On the other hand, associations between the physical component summary of the SF-36 and depression were only seen among the dialysis patients (OR = 0.906; P = 0.001)(Table 3).

**Table 3.** Results from the logistic regression analysis* (only factors associated with depression; P < 0.05)

Variables	Transplantation					Dialysis			
	ß	OR	95% CI	P-value		ß	OR	95% CI	P-value
Mental component summary	-0.080	0.923	0.85-0.99	0.03		-0.125	0.882	0.83-0.93	< 0.001
Physical component summary	- -	- -	- -	- -		-0.099	0.906	0.85-0.96	0.001

*Logistic regression model adjusted for age and sex.OR = odds ratio; CI = confidence interval.

### Characteristics of the patients with anxiety symptoms

Univariate analysis revealed that most of the transplantation patients with anxiety symptoms were women (P = 0.01) with higher CCI (P = 0.01) and lower scores in the SF-36 domains of bodily pain (P = 0.006), role physical (P = 0.01), role emotional (P = 0.01) and mental health (P ≤ 0.001), and in the mental component summary (P = 0.004) (Table 2). Comparedwith dialysis patients who did not show symptoms of anxiety, those who showed such symptoms tended to have brown or black skin color (P = 0.01), higher CCI (P = 0.001), higher numbers of hospital admissions (P = 0.008), histories of loss of vascular access over the last 12 months (P = 0.009) and lower scores in the SF-36 domains of role-physical (P = 0.02), bodily pain (P<0.001), general health status (P ≤ 0.001), vitality (P= 0.004), social functioning (P ≤ 0.001), role-emotional (P = 0.002) and mental health (P≤0.001), and in the physical component summary (P = 0.004) and mental component summary (P ≤ 0.001) (Table 2).

Logistic regression analysis showed that loss of vascular access over the last 12 months (OR = 3.672; P = 0.04), CCI (OR=1.578; P=0.01) and the physical component summary (OR=0.943; P=0.03) and mental component summary (OR=0.928; P=0.002) of the SF-36 were associated factors among dialysis patients(Table4).

**Table 4.** Results from the logistic regression analysis* (only factors associated with anxiety; P < 0.05)

Variables	Transplantation					Dialysis			
	ß	OR	95% CI	P-value		ß	OR	95% CI	P-value
Loss of vascular access	- -	- -	- -	- -		1.301	3.672	1.05-12.78	0.04
CCI	- -	- -	- -	- -		0.456	1.578	1.09-2.27	0.01
Mental component summary	- -	- -	- -	- -		-0.074	0.928	0.88-0.97	0.002
Physical component summary	- -	- -	- -	- -		-0.059	0.943	0.89-0.99	0.03

*Logistic regression model adjusted for age and sex. OR = odds ratio; CI = confidence interval; CCI = Charlson Comorbidity Index; loss of vascular access in the last 12 months.

### Severity of depression and anxiety symptoms

Patients scoring higher than 20 points in the BDI and BAI were diagnosed as presenting moderate to severe depression or anxiety. Logistic regression analysis revealed that poorer nutritional status (OR = 16.264; P = 0.02) and poorer general health status (OR = 0.961; P = 0.02) were associated with worsening of depression, whereas the presence of bodily pain (OR = 0.935; P = 0.004) and social functioning, as participation in some social activity at least once a month (OR = 0.081; P = 0.01), were associated with anxiety symptoms of greater severity (Table 5).

**Table 5.** Results from the logistic regression analysis* considering all patients (only factors associated with the severity of the symptoms of depression and anxiety; P < 0.05)

Variables	Depression (BDI score ≥ 20)					Anxiety (BAI score ≥ 20)			
	ß	OR	95% CI	P-value		ß	OR	95% CI	P-value
Bodily pain	- -	- -	- -	- -		-0.067	0.935	0.89-0.97	0.004
Social functioning	- -	- -	- -	- -		-2.516	0.081	0.01-0-13	0.01
SGA	2.789	16.264	1.34-196.26	0.02		- -	- -	- -	- -
General health status	-0.040	0.961	0.928-0.995	0.02		- -	- -	- -	- -

BDI = Beck Depression Inventory; BAI = Beck Anxiety Inventory; OR = odds ratio; CI = confidence interval. Social functioning = participation in some social activity at least once a month; suspected of being malnourished or severely malnourished according to the Subjective Global Assessment (SGA). *Logistic regression model adjusted for age and sex; combined analysis for dialysis and transplantation patients.

## DISCUSSION

In this study, the prevalence rates of symptoms of depression and anxiety were 31.2% and 27.9%, respectively, among the overall sample studied. Furthermore, depression affected approximately three times more dialysis patients than transplantation patients, whereas anxiety affected 1.5-times more dialysis patients.Depression was associated with the mental component summary of the SF-36 among both transplantation and dialysis patients. However, the physical component summary of the SF-36 only showed an association with dialysis. Anxiety was associated with loss of vascular access over the last 12 months and with the physical and mental component summaries of the SF-36 among dialysis patients. In the overall population studied, poorer nutritional status and poorer general health status were associated with worsening of depression, whereas low levels of recreational activities in daily living and presence of bodily pain were associated with anxiety symptoms of greater severity.

Symptoms of depression and anxiety are very common in cases of chronic health conditions, with higher prevalence among affected individuals than among the general population.^7^^,^^9^ The development of mental disorders can be influenced by patients’ social situation, such that the clinical condition of chronicity implies limitation or even loss of work capacity, financial deterioration and increased isolation in interpersonal relations.^33^

Research on end-stage renal disease has revealed anxiety and depression rates ranging from 12 to 60% and 10 to 70%, respectively.^7^^,^^8^^,^^9^^,^^10^^,^^12^^,^^13^^,^^14^^,^^15^ These percentages are subject to variations according to the features of specific studies, such as the diagnostic criteria, assessment tools and population characteristics.

The type of renal replacement therapy has been shown to be an important factor associated with mental health. In previous studies, transplantation patients were found to score lower than dialysis patients for both anxiety and depression.^34^^,^^35^^,^^36^ The mental health of hemodialysis and peritoneal dialysis patients is thought to be impacted to a greater extent than that of transplantation patients because of the strict routine of dialysis sessions, along with the countless restrictions that limit these individuals’ full participation in social, familial and productive activities. In contrast, kidney transplantation promotes greater wellbeing and freedom from dialysis and related restrictions and has a positive impact on self-perceived health.^1^^,^^34^^,^^35^^,^^36^^,^^37^^,^^38^^,^^39^^,^^40^

This phenomenon is particularly true for physical functioning, as shown by improved clinical parameters and nutritional status.^33^^,^^38^ Good physical functioning translates into positive changes in the lives of transplantation patients overall, including vitality and resumption of activities of daily living and interpersonal relationships, with resulting improvements in these individuals’ general emotional state. In a study on 80 renal transplantation patients, 75% reported having considerable improvement in their physical condition one and four years after the surgical procedure, which directly impacted their work and social activities.^37^

In this study, poorer quality of life was associated with depressive symptoms among transplantation and dialysis patients alike, whereas anxiety was associated with low quality of life in dialysis patients only. The relationship between mental disorders and quality of life is complex and needs to be discussed in a comprehensive manner. Despite advancements in renal replacement therapies, improved control over chronic kidney disease symptoms cannot prevent deterioration of quality of life. This has a significant impact on patient vitality and physical and mental capacity.^33^ Depression and anxiety not only interfere with the routine and habits of the individuals affected, but also impact self-perceived health and the ability to manage the many positive and negative aspects of life. Self-care skills of this nature are vital for improved clinical outcomes.^14^^,^^15^^,^^16^^,^^17^^,^^18^^,^^19^ Thus, the quality of life of end-stage renal disease patients is reduced in the presence of affective symptoms, which leads to poor clinical outcomes and decreased ability to face the demands of the disease and its treatment.

Negative correlations between emotional disorders and quality of life domains have been widely reported.^14^^,^^15^^,^^18^^,^^19^^,^^21^^,^^22^ Perales Montilla etal.^41^ compared the capacity of self-reported somatic symptoms and depression and anxiety for predicting quality of life among patients with chronic renal disease. Their results indicated that mood was a predictor of reductions in the physical and mental components of the SF-36, compared with the number and severity of physical symptoms.^41^ A cross-sectional study on 1,332 hemodialysis patients revealed that physical, psychological and social quality-of-life domains were negatively impacted by symptoms of depression and anxiety.^21^ In another study, depression was negatively correlated with all SF-36 scores among 105 patients undergoing peritoneal dialysis.^18^

The relationship between renal replacement therapy type and affective symptoms or quality of life differed between dialysis and transplantation patients in the present study. For dialysis patients, lower physical and mental component scores were associated with both conditions (depression and anxiety), while for transplantation patients, a relationship between mental component scores and depression was the only association found. Transplantation is the best alternative for replacement therapy, but the quality of life of transplantation patients is not comparable with that of the general population.^39^^,^^42^ Some studies have shown that, unlike physical quality-of-life domains, mental domains are not significantly affected by kidney transplantation.^43^

In a comparative evaluation of patients on hemodialysis and peritoneal dialysis and those who underwent renal transplantation, Fructuoso etal.^44^ showed that renal transplantation patients had higher values only in the physical domains. On the other hand, no significant differences were found in the mental domains between these groups of patients.^44^ Similar results were found in a study conducted by Czyżewski etal.,^33^ in which 47 transplanted patients had better outcomes in the physical domains of the SF-36, compared with 40 patients on dialysis, and no difference in the values was reported for the mental domains.^33^

Notably, transplantation patients do not fully regain pre-chronic kidney disease levels of function and remain chronic patients requiring complex ongoing treatment. Post-transplantation challenges arise, and these interfere with the reestablishment of quality of life (and therefore mental health), such as living with feelings of uncertainty regarding graft survival, potential graft rejection and hospitalization; adherence to strict drug regimens; dealing with the side effects of immunosuppressant medication and bodily image changes; and the need for constant surveillance and self-care.^37^^,^^38^^,^^39^^,^^42^

Among the dialysis patients of the present study, anxiety was associated with clinical status, as shown by the poorer scores for the physical and mental quality-of-life components, along with higher rates of comorbidities and loss of vascular access. The lack of additional factors associated with anxiety in the transplantation patient group can be explained by the fact that kidney transplantation promotes better health outcomes and greater freedom from treatment, compared with dialysis.^36^^,^^37^ Similar findings were described by Feroze etal.,^43^ who demonstrated that anxiety symptoms were connected with specific characteristics of dialytic treatment and comorbidities.^43^

Patients on hemodialysis or peritoneal dialysis are subjected to more onerous dialysis-related restrictions, have poorer physical parameters and experience more comorbidities and greater symptom burden. They therefore have a heavier burden of concerns and challenges, which arise from their various healthcare demands and needs. A cross-sectional study on 187 end-stage renal disease patients evaluated the symptom burden due to chronic kidney disease and the corresponding relationship with negative emotional states. It was concluded that psychological disturbances were associated with higher symptom burden and greater severity.^45^

Vascular access is one such challenge, particularly when an arteriovenous fistula (the most common and safest form of access for hemodialysis patients) is involved. The association between anxiety and loss of vascular access may be understood through considering the importance of this access for patient survival given that this forms the route through which effective dialysis can be performed. Alongside clinical complications, loss of vascular access gives rise to negative emotions such as anguish and discomfort. Chronic pain and limitations regarding several aspects of life often also form part of this picture, thereby contributing towards development and persistence of anxiety disorders.^43^

Several factors are known to affect the prognosis and severity of anxiety and depression, such as individual characteristics, genetic load, stressful life events, concurrent mental disorders and health status.^46^ In the present study, greater severity of depressive symptoms was associated with worse general health status and poorer nutritional status.

The relationship between nutritional status and the severity of depressive symptoms needs to be appreciated from different perspectives. One potential explanation for this relationship is the negative impact of affective disorders on eating behavior. However,these disorders may be concurrent with ongoing nutritional deficits and underlying disease progression.^47^

The role of mental health in healthy behaviors also needs to be emphasized. This includes adequate food intake, since depression is known to interfere with eating habits and may lead to either increased or decreased appetite.^44^ Additionally, depression has been positively correlated with undernourishment and poorer levels of hemoglobin, ferritin and albumin, in some end-stage renal disease studies.^47^^,^^48^

The presence of bodily pain and less frequent participation in recreational activities were associated with greater severity of anxiety symptoms in the present study. Anxiety has been correlated with complaints of pain. Some studies have shown that patients with chronic pain had elevated levels of concern, tension and nervousness with regard to their illness and their general clinical condition, which influenced their perception of the painful experience.^49^ On the other hand, states of pain, whether acute or chronic, favor psychological manifestations and become a factor in increasing the incidence of mood and anxiety disorders among these patients, compared with the general population.^49^^,^^50^

Considering that chronic kidney disease increases the risk of having pathological conditions such as diabetes mellitus, neurological conditions, bone diseases and vascular diseases, patients undergoing renal replacement therapy are more likely to experience different types of pain of variable intensity and in a variety of locations. These patients’ types of pain are associated not only with their pathological condition but also with the intercurrences and specificities of the renal treatment itself.^50^

A cross-sectional study on 205 patients on dialysis showed that there was higher prevalence of mental disorders among patients with moderate or severe chronic pain than among those with mild or no pain. Severe irritability and anxiousness, and inability to cope with stress, were also more common among patients with pain than among those without pain.^51^ Overall, chronic kidney disease patients participate less in recreational activities after they have started to undergo renal replacement therapies.^52^ Although their reduced engagement in social activities may be partly due to their clinical status, the type of renal replacement therapy also needs to be considered, as shown by the lower scores among patients undergoing dialysis.^35^

A systematic review of the literature that examined studies comparing the level of engagement in activities of daily living among adult chronic kidney disease patients who underwent different types of therapy concluded that transplantation patients experienced greater levels of social inclusion, while hemodialysis and peritoneal dialysis patients did not differ significantly in this regard.^52^ The benefits of recreational activities for emotional wellbeing and quality of life include feelings of satisfaction, perceived freedom of choice and engagement in and expansion of social networks.

Although the data in our study were derived from a cross-sectional follow-up study on participants from another cohort that had been established in 2006, this work has made a contribution to the scarce scientific literature. Nonetheless, at the end of the follow-up, 507patients were censored, and there were 449 deaths, representing 40.06% of the initial sample. A high mortality rate is expected among end-stage renal patients, especially in the first years of dialysis, because of several factors such as advanced age, diabetes mellitus, the underlying cause of chronic kidney disease and residence in cities with worse developmental rates.^52^^,^^53^^,^^54^^,^^55^ Accordingly, the patients participating in our study were the ones who survived and therefore were in a better clinical and emotional condition.

Some limitations of the present study need to be considered. Firstly, simultaneous occurrence of end-stage renal disease and affective disorder symptoms needs to be considered. Coexistence of symptoms associated with both the uremic state and depressive mood, such as fatigue, reduced appetite, memory impairment and irritability, may occur.

Secondly, despite broad application and validation for end-stage renal disease patient populations, Beck’s inventories have limitations that may interfere with making diagnoses of depression and anxiety, such as use of self-report questionnaires and inclusion of somatic symptoms that are not exclusive to emotional disorders. Additionally, given that uremic parameters and graft function were not considered in the present study, associations between affective symptoms and actual renal function status could not be established. Transplantation patients with impaired graft function usually have higher levels of depression or anxiety because they face the fear of having to start to undergo dialysis again.

Thirdly, the results of the present study were derived from a sub-cohort with a 10-year follow-up. Therefore, survival biases may not have been eliminated, given that the participants potentially reflected those with better health status. Among dialysis patients, long-term survivors may exhibit less evidence of depression or anxiety or may experience severely affected mental health when they have no options for transplantation.

Fourthly, the low number of patients in the renal transplantation group may have hindered possible detection of an association between anxiety and quality of life. This evaluation could not be made in the present study.

Lastly, given the cross-sectional study design, no causal links could be established, and the progression of depression and anxiety symptoms over time could not be measured. For these factors to be measured, longitudinal approaches are required.

## CONCLUSION

This study revealed that depression and anxiety are common conditions among chronic kidney disease patients and that they occur more frequently among those undergoing dialysis than among those undergoing transplantation. Lower quality-of-life scores were associated with symptoms of depression in both types of renal replacement therapy. Presence of comorbidities, loss of vascular access and worse quality of life were associated with anxiety symptoms among dialysis patients, whereas none of these factors was associated with anxiety symptoms among transplantation patients. Treatment of affective disorders needs to be effectively included within the routine care provided for chronic kidney disease patients and should be maintained across the continuum of care. Further investigations are warranted to identify major risk factors and design better interventions for management, control and prevention.
